# Transection Speed and Impact on Perioperative Inflammatory Response – A Randomized Controlled Trial Comparing Stapler Hepatectomy and CUSA Resection

**DOI:** 10.1371/journal.pone.0140314

**Published:** 2015-10-09

**Authors:** Christoph Schwarz, Daniel A. Klaus, Bianca Tudor, Edith Fleischmann, Thomas Wekerle, Georg Roth, Martin Bodingbauer, Klaus Kaczirek

**Affiliations:** 1 Dept. of Surgery, Div. of General Surgery, Medical University of Vienna, Vienna, Austria; 2 Section of Transplantation Immunology, Dept. of Surgery, Medical University of Vienna, Vienna, Austria; 3 Dept. of Anesthesiology, General Intensive Care and Pain Medicine, Medical University of Vienna, Vienna, Austria; The Chinese University of Hong Kong, HONG KONG

## Abstract

**Background:**

Parenchymal transection represents a crucial step during liver surgery and many different techniques have been described so far. Stapler resection is supposed to be faster than CUSA resection. However, whether speed impacts on the inflammatory response in patients undergoing liver resection (LR) remains unclear.

**Materials and Methods:**

This is a randomized controlled trial including 40 patients undergoing anatomical LR. Primary endpoint was transection speed (cm^2^/min). Secondary endpoints included the perioperative change of pro- and anti-inflammatory cytokines, overall surgery duration, length of hospital stay, morbidity and mortality.

**Results:**

Mean transection speed was significantly higher in patients undergoing stapler hepatectomy compared to CUSA resection (CUSA: 1 (0.4) cm^2^/min vs. Stapler: 10.8 (6.1) cm^2^/min; p<0.0001). Analyzing the impact of surgery duration on inflammatory response revealed a significant correlation between IL-6 levels measured at the end of surgery and the overall length of surgery (p<0.0001, r = 0.6188). Patients undergoing CUSA LR had significantly higher increase of interleukin-6 (IL-6) after parenchymal transection compared to patients with stapler hepatectomy in the portal and hepatic veins, respectively (p = 0.028; p = 0.044). C-reactive protein levels on the first post-operative day were significantly lower in the stapler cohort (p = 0.010). There was a trend towards a reduced overall surgery time in patients with stapler LR, especially in the subgroup of patients undergoing minor hepatectomies (p = 0.020).

**Conclusions:**

Liver resection using staplers is fast, safe and suggests a diminished inflammatory response probably due to a decreased parenchymal transection time.

**Trial Registration:**

ClinicalTrials.gov NCT01785212

## Introduction

Hepatic resection is the treatment of choice for several malignant and benign liver diseases. [1] Parenchymal transection represents a crucial part in liver surgery and many different techniques have been described with comparable results in outcome. [2] Transection speed and blood loss during resection, are the two cornerstones that adversely affect the postoperative course of patients after hepatic surgery. [3] Thus, fast and safe techniques are required, in order further improve patient’s outcome.

Parenchymal transection with an ultrasonic dissector such as the cavitron ultrasonic surgical aspirator (CUSA) is one of the most commonly used techniques in liver surgery providing for safe and exact dissection. [4] However, the relatively long transection time is one disadvantage, which negatively impacts on overall surgery duration. Recently the technique of stapler hepatectomy is becoming more and more popular [5] as several retrospective trials have shown the efficacy and safety of stapler transection. [6, 7] There exist only two randomized controlled trials evaluating stapler hepatectomy in comparison to clamp-crush technique (CRUNCH trial) [8] and ultrasound dissection [9]. Thus so far, data available showing the true benefit of stapler hepatectomy in liver resection (LR) is limited.

It has been shown that the release of cytokines, chemokines and stress hormones is associated with surgical trauma and correlates with postoperative outcome and organ dysfunction. [10, 11] Especially in liver surgery, pro- and anti-inflammatory cytokines orchestrate a wide range of physiological and pathological pathway, ranging from inflammation and organ failure to liver regeneration. [12–14] Hepatic resection is usually performed under low central venous pressure anesthesia (LCVP) in order to reduce bleeding. [15, 16] However, LCVP is generally associated with arterial hypotension [17], thus a systemic mal-perfusion in major surgery might further aggravate an inflammatory response.

Aim of this study was to compare stapler hepatectomy with CUSA resection regarding transection speed and its impact on inflammatory response. We hypothesized that a faster parenchymal transection would lead to a decrease in inflammatory cytokines in patients undergoing LR.

## Materials and Methods

This is a single-blinded, prospective, randomized controlled trial, which was conducted in patients undergoing LR at the Department of Surgery at the Medical University of Vienna between August 2013 and December 2014 (see S1 Protocol). Patients were randomized in a 1:1 ratio to CUSA resection or stapler hepatectomy. The study was reviewed and approved by the institutional review board of the Medical University of Vienna (IRB nb.: 1829/2012) and registered at the clinicaltrials.gov database (ID nb: NCT01785212). Written informed consent was obtained from all participants.

### Patient population

Patients older than 18 years undergoing an elective hepatic resection including ≥ two segments of the liver, and expected feasibility of stapler hepatectomy and CUSA resection based on preoperative imaging were eligible. Exclusion criteria were Hepatitis B, C or HIV infection, autoimmune disease, inflammatory bowel disease or pregnancy. Preoperative liver function was assessed by indocyanine green clearance test (ICG) as described previously. [18]

Randomization was performed intraoperative after exploration of the abdominal cavity for resectability by using an online software (Randomizer for Clinical Trials 1.8.1.; Institute for Medical Informatics, Statistics and Documentation at Medical University of Graz, Graz, Austria).

### Surgical technique

Stapler hepatectomy was performed by crushing the liver parenchyma with a Pean clamp and subsequently divided using Covidien Endo-Gia™ Ultra Handle Short Staplers and Endo Gia™ TRI staple with 60 mm or 45 mm AVM/AMT loading units (Covidien, Brunn/Gebirge, Austria). In the CUSA group the liver parenchyma was divided along the transection line by CUSA (Cavitron ultrasonic aspirator; Valleylab, Boulder, CO) and bipolar forceps in a two surgeon technique. Vessels of less than 2 mm in diameter were coagulated with bipolar forceps. The remaining vessels were clipped or ligated. Hepatic veins and portal pedicles were clamped and suture ligated. The type of LR was defined as major and minor resections according to the IHPBA Brisbane 2000 nomenclature (≤2 segments: minor; >2 segments: major). [19]

### Study endpoints

Primary endpoint was the transection time of the liver normalized to the transection surface. The transection phase started with opening the liver parenchyma after the transection line had been marked by electrocautery. It ended after complete division of the liver parenchyma. Time was measured by a blinded anesthesiologist and expressed in seconds. The cut surface of the resected liver was photographed together with a 4 cm^2^ reference scale in an exact 90° angle. The area of the liver transection surface was calculated in cm^2^ by setting the measured pixels of the cut surface in relation to the reference scale using ImageJ software 1.48v (Rasband, NIH, USA). The transection speed was expressed in cm^2^/min.

Secondary endpoints were perioperative cytokine concentrations in correlation to transection speed including interleukin-6 (IL-6), IL-8, IL-10, TNFa. The increase of IL-6 caused by parenchymal transection was measured in the portal vein, hepatic vein and systemically (post-resection–pre-resection). C-reactive protein (CRP) levels were monitored perioperative and followed for the first six days after LR. Morbidity and mortality was assessed according to the Clavien-Dindo classification [20] within 30 days follow-up. Furthermore, operation time, length of intensive care unit and hospital stay were analyzed.

### Blood sampling

Blood samples were obtained pre-surgery (systemic), pre-resection (systemic, portal vein, hepatic vein), post-resection (systemic, portal vein, hepatic vein), post-surgery (systemic) and on post-operative days (POD) 1 and 3 (systemic). Blood samples were collected in pre-chilled Z Serum clot activator containing vacuum tubes. After centrifuging the samples at 1400 RPM (rounds per minute) for 10 min at 4°C, serum was stored at -80°C. Cytokine levels were determined by using RayBio® Quantibody Human Th1/Th2 Array 1 (RayBiotech, Norcross, GA, USA). Cytokine concentrations were assessed by RayBiotech Inc. by using their Quantibody service.

### Statistical analysis

Sample size was calculated for 20 patients per group based on the expected difference (3.9 ± 0.3 cm^2^/min (SEM) for clamp crush and 2.3 ± 0.2 cm^2^/min for CUSA resection) in transection speed observed in a prospective study evaluating different techniques including clamp-crush resection with vascular occlusion (comparable to speed of stapler hepatectomy) and CUSA resection [21] to achieve an alpha of 0.05 and a power of 80%, including a potential drop out rate of 15%.

Statistical analysis was performed using GraphPad Prism, version 6 (GraphPad Prism Software®, La Jolla, CA). Metric data were expressed as means with SD or median with interquartile range (Q1-Q3) and analysis was performed with the Mann-Whitney U test or an unpaired t-test as indicated. The individual change in cytokines was calculated using a paired t-test. Categorical values were compared with Fishers-exact test or a chi-square test. Correlation between length of surgery and systemic IL-6 concentration was tested with the Spearman correlation coefficient. A p-value of <0.05 was determined as the threshold for significance.

## Results

**Fig 1:**
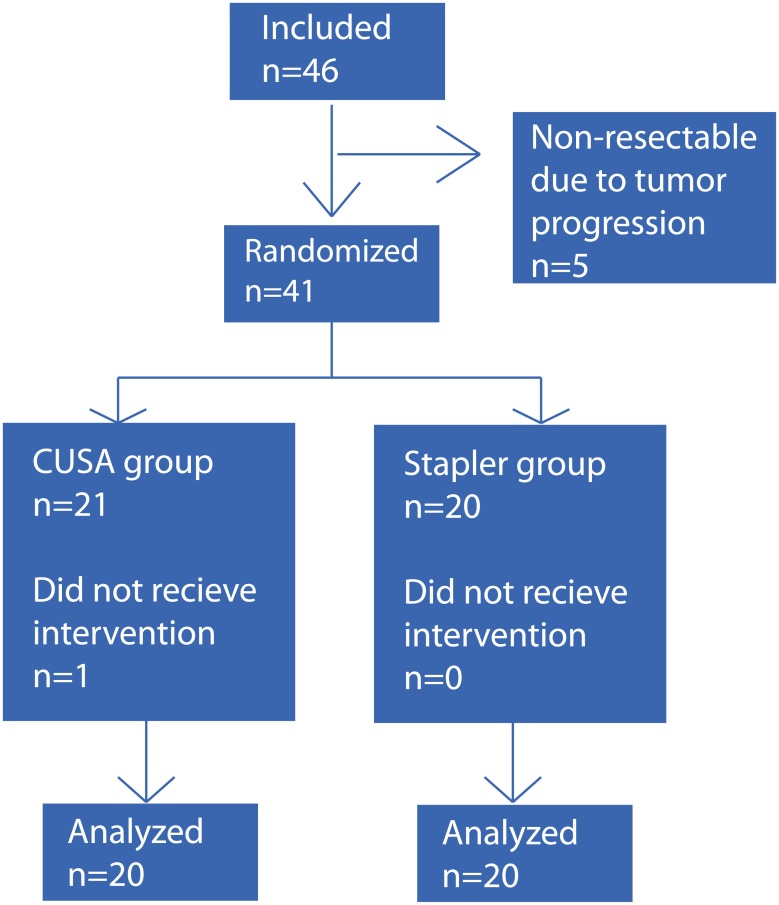
Enrollment and randomization. Forty-six patients were enrolled for this study. In five patients liver resection was not performed due to tumor progression. The remaining 41 patients were randomized intraoperative and assigned to CUSA resection (n = 21) or stapler hepatectomy (n = 20). One patient from the CUSA group was excluded after randomization, as the patient emerged to be non-resectable.

Between August 2013 and December 2014 forty-six patients were eligible for the study and signed informed consent. Five patients were excluded intraoperative prior to randomization due to unexpected tumor progression. The remaining 41 patients were randomized; one patient was excluded after randomization due to non-resectable liver disease (Fig 1).

### Patient characteristics

Baseline characteristics were broadly comparable between the CUSA resection group and stapler hepatectomy study group. The main indication for LR was colorectal liver metastasis (45% vs. 35%, p = 0.748) followed by echinococcus cyst(s) (15% vs. 10%, p = 1.000) and hepatocellular carcinoma (10%, vs. 15%; p = 1.000). There was no significant difference in gender (male: 55% vs. 50%; p = 1.000), age (61.6 (49.5–67.9) vs. 60.5 (41.3–71.3); p = 0.914) or American Society of Anesthesiologists (ASA) grade (p = 0.963) in patients with CUSA resection and patients with stapler hepatectomy, respectively (Table 1). There was a trend towards a higher rate of major hepatectomies in patients undergoing stapler resection though the observed difference did not reach statistical significance (55% vs. 70%, p = 0.515) (Table 2). In two patients (each group one) two anatomical resections were performed simultaneously (right hepatectomy + segment III and segment II/III + VI/VII). Transection time and resection surface was added to calculate transection speed in both patients.

**Table 1:** 

Patients charcteristics	CUSA hepatectomy n = 20	Stapler hepatectomy n = 20	Overall n = 40	p value
Age [years], median (Q1-Q3)	61.6 (49.5–67.9)	60.5 (41.3–71.3)	60.5 (44.7–71.3)	0.855^a^
Sex [male], n (%)	11 (55)	10 (50)	21 (52.5)	1.000^b^
**Cause for liver resection, n (%)**				
CRCLM	9 (45)	7 (35)	16 (40)	0.748^b^
Echinococcus	3 (15)	2 (10)	5 (12.5)	1.000^b^
HCC	2 (10)	3 (15)	5 (12.5)	1.000^b^
Adenoma	1 (5)	2 (10)	3 (7.5)	1.000^b^
Others	5 (25)	6 (30)	11 (27.5)	1.000^b^
Preoperative chemotherapy, n (%)	9 (45)	9 (45)	18 (45)	1.000^b^
**ASA, n (%)**				0.963^a^
Grade I	3 (15)	4 (20)	7 (10)	
Grade II	7 (35)	6 (30)	13 (32.5)	
Grade III	10 (50)	10 (50)	20 (50)	
Grade IV	0	0	0	
**ICG clearance**				
PDR, median (Q1-Q3)	25.2 (21.3–27)	24.9 (17.5–29.9)	25.2 (19.4–28.3)	0.951^a^
R15, median (Q1-Q3)	2.3 (1.7–4.1)	2.4 (1.1–7.2)	2.3 (1.5–5.5)	0.911^a^

^a^ Mann-Whitney U test,

^b^ Fishers-exact test

**Table 2:** 

Intraoperative data	CUSA hepatectomy n = 20	Stapler hepatectomy n = 20	Overall n = 40	p value
**Type of resection, n (%)**				0.515^a^
major hepatectomy	11 (55)	14 (70)	25 (62.5)	
minor hepatectomy	9 (45)	6 (30)	15 (37.5)	
Transection speed [cm2/s], mean (SD)	1 (0.4)	10.8 (6.1)	5.9 (6.6)	<0.0001^b^
**Surgery duration, median (Q1-Q3)**	230 (191.5–278.5)	197.5 (147.3–238.8)	215.5 (163.8–271.3)	0.151^c^
minor hepatactomy	210 (160–270)	145 (110–165)	160 (127.5–230)	0.012^c^
major hepatactomy	270 (222.5–280)	218 (192.5–268.8)	230 (200–278)	0.492^c^
Intraoperative requirement for blood products, n (%)	1 (5)	0	1 (2.5)	1.000^a^

^a^ Fishers.exact test,

^b^ unpaired t-test,

^c^ Mann-Whitney U test

### Outcome

Transection speed, the primary endpoint, was significantly faster in patients undergoing stapler resection compared to the CUSA control group (CUSA: 1 (0.4) cm^2^/min vs. Stapler: 10.8 (6.1) cm^2^/min; p<0.0001). Even though this resulted in a shorter median length of surgery, the observed difference did not reach statistical significance (230 (191.5–278.5) min vs. 197.5 (147.3–238.8); p = 0.104). However, analyzing the subtypes of LR, we observed a significantly reduced operation time in patients with minor LR with stapler hepatectomy compared to CUSA LR (210 (160–270) min vs. 160 (127.5–230) min; p = 0.012) (Table 2).

Post-operative complications assessed by Dindo classification were low and comparable in both cohorts (p = 0.305) (Table 3). In particular there was no difference in post-operative bleeding, bile leakage or infection. One patient from the stapler cohort died due to liver dysfunction on the 26^th^ POD.

**Table 3:** 

Postoperative Outcome	CUSA hepatectomy n = 20	Stapler hepatectomy n = 20	Overall n = 40	p value
Positive resection margin (R1), n	0/12	0/15		1.000^a^
**Clavien-Dindo Grade, n (%)**				0.305^b^
I	2 (10)	1 (5)	3 (7.5)	
II	1 (5)	1 (5)	2 (5)	
IIIa	2 (10)	2 (10)	4 (10)	
IIIb	1 (5)	3 (15)	4 (10)	
IVa	0	1 (5)	1 (2.5)	
IVb	0	0	0	
V	0	1 (5)	1 (2.5)	
Hospital stay duration, median (Q1-Q3) §	9.5 (8–11)	12 (11–20.3)	11 (8.8–13.3)	0.009^b^
Time on ICU, median (Q1-Q3)	1 (1–2)	1 (1–2)	1 (1–2)	0.373^b^

^§^ in case of death (n = 1) hospital stay was imputed with 78

^a^ Fishers-exact test,

^b^ Mann-Whitney U test

The median length of intensive care unit (ICU) stay after LR was similar between both groups (1 (1–2) vs. 1 (1–2); p = 0.373). However, a longer hospital stay was observed in patients undergoing stapler resection (9.5 (8–11) vs. 12 (11–20.3) days; p = 0.009).

### Inflammatory parameters

**Fig 2:**
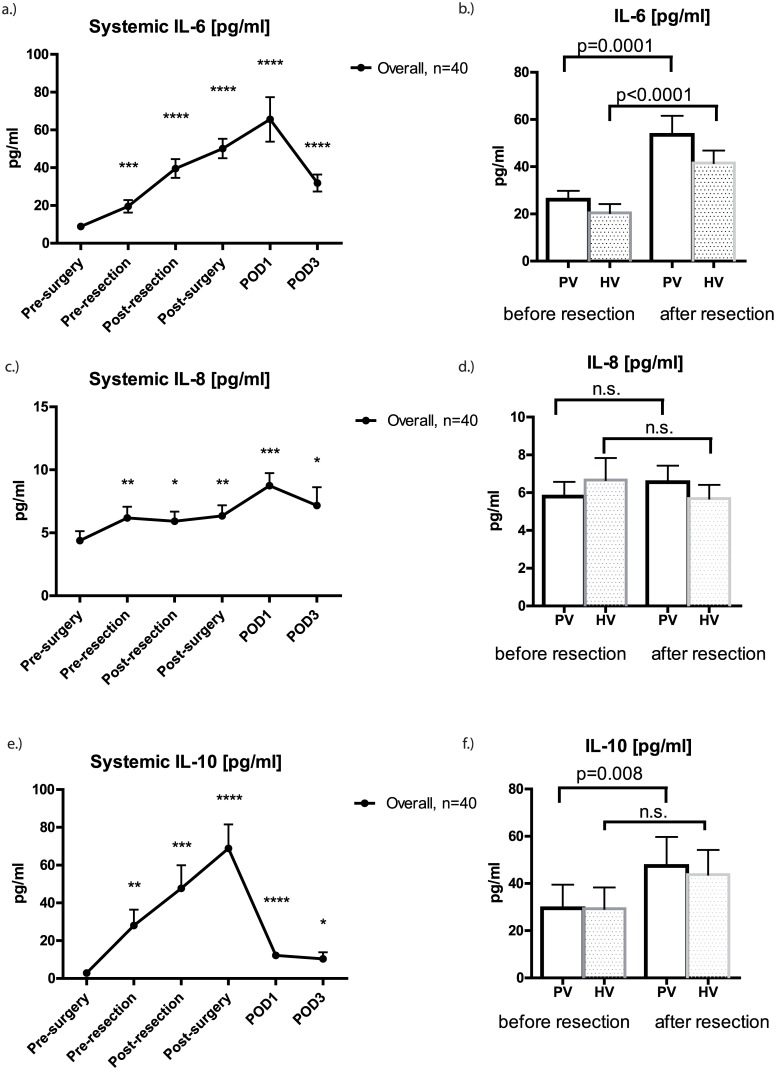
Perioperative alteration of pro- (IL-6, IL-8) and anti-inflammatory cytokines (IL-10). Systemic IL-6 **(A)**, IL-8 **(C)** and IL-10 **(E)** levels significantly increased perioperatively compared to baseline values. Graphs show mean cytokine concentrations [pg/ml] ± SEM. There was a significant increase in IL-6 during liver resection in the portal vein (PV; p = 0.0001) and hepatic vein (HV; p<0.0001) respectively **(B)**. Similar to that IL-10 increased significantly in the PV (p = 0.008) but not HV **(F)**. IL-8 levels measured in the in and out flow remained virtually unchanged (**D)**. Statistical significance compared to baseline values is abbreviated with * (p = 0.010–0.050), ** (p = 0.001–0.010), *** (p = 0.0001–0.001) or **** (p < 0.0001).

Overall, there was significant increase in systemic IL-6, IL-8 and IL-10 perioperative compared to baseline values. These cytokines peaked after skin closure (IL-10) or on post-operative day 1 (IL-6, IL-8) and declined after the peak. Mean IL-6 increased significantly in the portal vein (PV: 26 (3.7) pg/ml vs. 53.5 (8) pg/ml; p = 0.0001) and hepatic vein (HV: 20.5 (3.7) pg/ml vs. 41.6 (5.3) pg/ml; p<0.0001). Similar to that, IL-10 increased significantly in the PV (29.5 (10) pg/ml vs. 47.5 (12.2) pg/ml; p = 0.008) but not HV (29.3 (9) pg/ml vs. 43.8 (10.4) pg/ml; n.s.). Notably, IL-8 levels measured in the in- and outflow of the liver remained stable during parenchymal transection (Fig 2).

**Fig 3:**
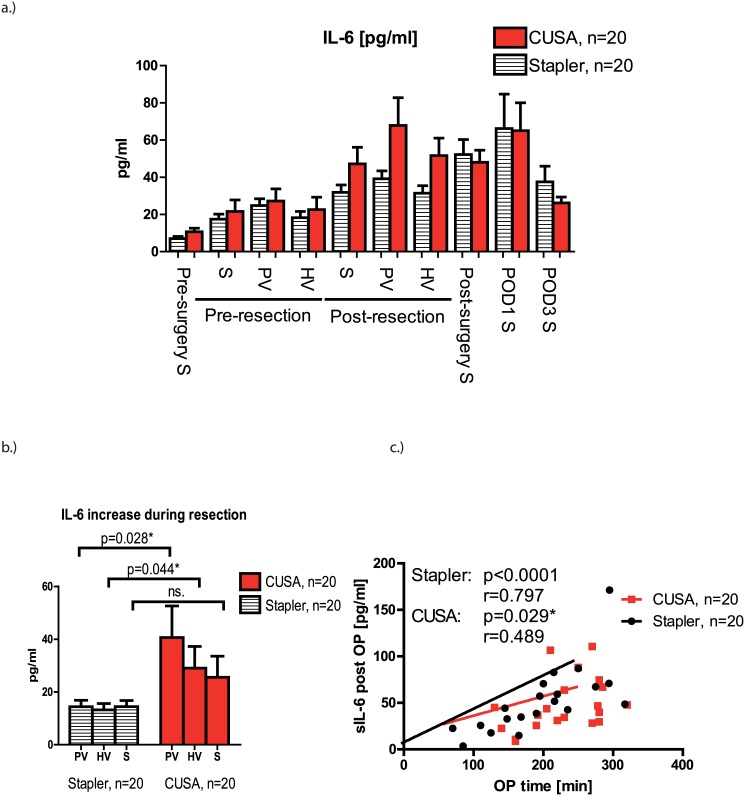
Stapler liver resection leads to a decreased IL-6 response compared to CUSA transection. **(A)** IL-6 production was numerical higher in patients undergoing CUSA resection, though the observed difference did not reach statistical significance. Cytokines levels are shown as means ± SEM. **(B)** Calculating the specific IL-6 response during liver resection revealed a significantly higher increase in the portal vein (PV) (p = 0.028) and hepatic vein (p = 0.044) but not systemically in patients in the CUSA group. **(C)** There was a strong correlation between systemic IL-6 measured immediately after skin closure and length of operation (CUSA: p = 0.029, r = 0.489; Stapler: p<0.0001, r = 0.797; Overall: p<0.0001, r = 0.6188).

**Fig 4:**
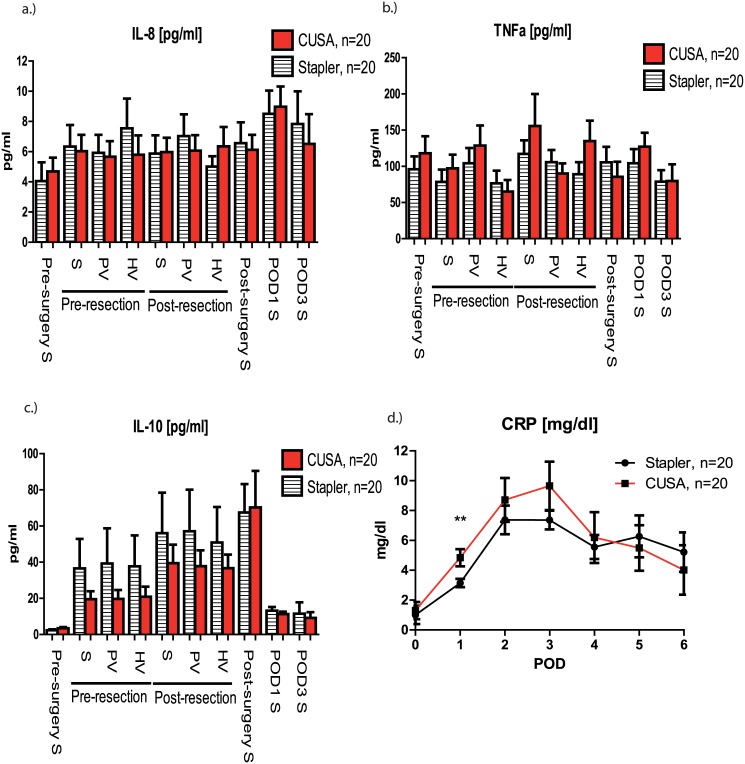
Perioperative course of IL-8, TNFa, IL-10 and C-reactive protein (CRP). There was no significant difference between both groups in perioperative IL-8 **(A)**, TNFa **(B)** or IL-10 **(C)** levels. CRP measured on the first postoperative day (POD) was significantly higher in patients undergoing CUSA resection than in patients with stapler hepatectomy (3.1 (0.3) vs. 4.8 (0.5); p = 0.010). Graphs show mean values ± SEM.

Analyzing both groups separately revealed that systemic IL-6 was similar in patients undergoing CUSA and stapler resection, respectively (Fig 3a). The specific IL-6 increase during parenchymal transection was significantly higher in patients with CUSA LR in the portal vein (40.6 (11.9) pg/ml vs. 14.4 (2.4) pg/ml; p = 0.026), hepatic vein (29 (8.2) pg/ml vs. 13.2 (2.4) pg/ml; p = 0.042) but not systemically (25.6 (8) pg/ml vs. 14.5 (2.3) pg/ml; p = 0.352) (Fig 3b). Investigating the impact of surgery duration on inflammatory response revealed a significant correlation between IL-6 levels measured at the end of surgery after skin closure and the overall length of surgery (CUSA: p = 0.029, r = 0.489; Stapler: p<0.0001, r = 0.797; Overall: p<0.0001, r = 0.6188) (Fig 3c). Notably, the perioperative course of IL-8, TNFa and IL-10 was comparable between CUSA resection and stapler hepatectomy, respectively (Fig 4a, 4b and 4c).

Finally, CRP levels, a marker for systemic inflammation, reached a peak on the third POD.

Significantly higher CRP levels measured on the first POD in patients with CUSA LR (4.8 (0.6) mg/dl vs. 3.1 (0.3) mg/dl; p = 0.010) (Fig 4d).

## Discussion

While LR using staplers has been shown to be both safe and feasible compared to other transection techniques, data supporting a benefit for patients undergoing stapler hepatectomy remain scarce. [5–9] Herein, we show for the first time that stapler LR results in both, an increased transection speed compared to LR with CUSA and a favorable influence on perioperative inflammatory response.

Transection speed, the primary endpoint of the study, was significantly higher in patients with stapler hepatectomy compared to patients undergoing conventional CUSA resection. These results are similar to those noted in the CRUNSH trial, which demonstrate a lower parenchymal transection time for stapler resections in contrast to the clamp-crush technique. [8] Savlid et al reported in a randomized controlled trial comparing stapler LR with CUSA hepatectomy similar results between both approaches. Interestingly, in contrast to our findings, they report only a trend towards shorter transection- and operating time that did not reach statistical significance. [9]

Prolonged parenchymal transection obviously may prolong anesthesia with low central venous pressure, which may be a cause for systemic hypoperfusion. [17] Tamion et al observed that gastric mucosal acidosis, a marker for splanchnic hypoperfusion, has been strongly associated with TNFa and IL-6 levels in critically ill patients with septic shock. [22] In another prospective study it has been suggested that, on the other side, a preserved splanchnic perfusion may reduce gut-related inflammatory response leading to a reduction in pro-inflammatory cytokines. [23] Thus, decreasing the time under LCVP, and thereby decreasing a potential organ malperfusion, may also lead to a decreased inflammatory response from the splanchnic region. In the present study, systemic perioperative IL-6, IL-8 and IL-10 levels were significantly increased during surgery compared to baseline values. Although the initial pattern of IL-6 release was similar in both groups, there was a significantly higher increase of IL-6 release in patients with CUSA LR in the portal- and hepatic vein respectively. Furthermore there was a clear association between length of surgery and IL-6 production. However, in spite of a lower increase in IL-6 during resection, cytokine levels at the end of surgery and on POD 1 and 3 were virtually identical in both groups. These results suggest that other factors than the transection technique itself predominantly impact on systemic IL-6 levels.

CRP is an acute phase protein that is produced by the liver in response to IL-6. [24] The timing of peak response usually occurs between the first and third POD and correlates with the magnitude of operative injury and procedure. [25] It has been suggested that after liver surgery, a higher CRP response is associated with a poorer prognosis. [26] Thus, the observation that CRP levels were significantly lower on first POD in patients undergoing stapler resection is further supporting our assumption that fast parenchymal transection using staplers provides for an improved inflammatory profile.

Even though we noted a significantly lower length of operation in patients undergoing minor resection, there was only a trend towards shorter surgery duration in patients with major resections. Reasons remain not fully understood but may partly be explained by a longer liver mobilizing time during major LR. Thus, the proportion of parenchymal transection time in overall operation duration is lower in patients with major resections than in patients undergoing minor LR.

Regarding surgical outcome, comparable results were observed in both groups. Length of ICU stay as well as incidence of complications was low in both groups respectively, similar to results from other studies evaluating stapler LR. [8, 9] However, LOS was significantly longer in patients with stapler LR. There are two potential explanations for these findings. Firstly, patients were not stratified during randomization according to the type of hepatic resection, so the proportion of patients undergoing major LR (more severe surgery) was numerically higher in the stapler cohort. Secondly, discharge from hospital is highly variable in our department depending from patients’ social network and support at home, and thus may differ greatly between individuals.

To conclude, LR using staplers is significantly faster than LR using CUSA. The increased speed results in a reduced release of IL6 during parenchymal transection and lower CRP levels on the first POD. Whether the reduced surgical stress response translates into a clinical benefit for patients undergoing hepatic resection remains a substance for future clinical trials.

## Supporting Information

S1 CONSORT Checklist CONSORT 2010 checklist. (DOC)

S1 Protocol Study protocol. (DOC)
