# Supplementary material for: Transection Speed and Impact on Perioperative Inflammatory Response – A Randomized Controlled Trial Comparing Stapler Hepatectomy and CUSA Resection
Source: PLoS One. 2015 Oct 9;10(10):e0140314. doi: 10.1371/journal.pone.0140314 (PMC4599945; doi:10.1371/journal.pone.0140314)
Supplement: S1 Protocol — (DOC) [file pone.0140314.s002.doc]

| Background and Rationale |
| --- |
| Many different techniques of parenchymal transection are used in hepatic surgery. In a systematic review, there were no significant differences in morbidity (including bile leak), mortality, routine markers of liver parenchymal injury or dysfunction and length of hospital stay irrespective of the method used for parenchymal transection. This Cochrane review analyzed studies comparing the following transection devices: CUSA (cavitron ultrasound surgical aspirator) versus clamp-crush (two trials); radiofrequency dissecting sealer versus clamp-crush (two trials); sharp dissection versus clamp-crush technique (one trial); and hydrojet versus CUSA (one trial). The clamp-crush technique appeared to have the lowest blood loss and lowest transfusion requirements compared to the other techniques .  However, even in specialized centers morbidity and mortality rates of hepatic resections are still in the range of 45% and 3% respectively  and uncertainty persists regarding the optimal technique of transection. Local experience seems to be the most important factor for the choice of the transection method. An innovative technique is stapler hepatectomy using Covidien Endo-Gia™ Ultra Handle Short Staplers and Endo Gia™ TRI staple 60mm or 45 mm AVM/AMT loading units (Covidien). A randomized controlled trial (CRUNSH trial) to evaluate the intraoperative blood loss of stapler hepatectomy compared to the clamp-crushing technique is currently under way .  The CUSA technique is well established in many centers including ours with excellent morbidity and mortality rates . However, it has been shown that CUSA has a longer transection speed than the clamp-crush technique (with vascular occlusion). The investigators of the CRUNSH trial hypothesize that stapler hepatectomy technique might also be comparable or more favorable to clamp-crushing regarding transection time with the advantage of avoiding vascular occlusion. Therefore stapler hepatectomy should also be faster than CUSA.  It has been shown that the release of cytokines, chemokines, and stress hormones correlates with postoperative infection and organ dysfunction . Chemokines are critically involved in the process of leukocyte recruitment and activation in the liver. Major surgery causes inflammation reflected in the production of pro-inflammatory cytokines . In various studies IL-6, for instance, was a valid predictor for post-operative sepsis , complications or mortality . Besides, the levels of these cytokines are expected to correlate with the degree of surgical trauma. Therefore differences in cytokine levels between the two study groups will be assessed, including pro- (INF-γ, IL-1β, IL-5, IL-6, IL12p70, TNFα) and anti-inflammatory (IL-4, IL-10, IL-13) cytokines.  Monocyte chemotactic protein-1 (MCP-1) production is elevated in Kupffer cells following ischemia / reperfusion in response to free radicals and neutrophil elastase, as well as in animal oxidative liver injury models (e.g. carbon tetrachloride)  Macrophage inflammatory protein-3-alpha (MIP3-alpha) is constitutively expressed in the liver. It is strongly chemotactic for cytokine-stimulated neutrophils, immature dendritic cells and memory/effector T and B lymphocytes by utilizing chemokine receptor (CCR) 6.  sCD163 (soluble haemoglobin scavenger receptor) is a novel marker of activated macrophages, like neopterin it can be determined in serum or plasma.  The effect of the transection speed in respect to chemokine release has never been investigated. We hypothesize that a shorter transection time leads to a reduced release of these molecules potentially resulting in improved postoperative outcome.  Additionally the interaction between adaptive and innate immunity plays a significant role in liver ischemia-reperfusion (I/R) injury . Notably, activation of T cells in the absence of TCR ligation seems to be a predominant factor in the initial phase of I/R injury . Therefore as a pilot study, peripheral T cell subsets (including naïve T cells, effector and central memory T cells, regulatory T cells, early activated T cells) will be determined by flow cytometry in a subgroup of study patients (i.e. patients undergoing hepatic resection for other than oncological reasons).  The supposedly slower technique of CUSA resection shall therefore be compared with the novel technique of stapler hepatectomy. |

| Objective |
| --- |
| Primary objective:Transsection speed (cm^2^/min) |
| **Secondary objectives**:   | - Perioperative chemokine concentrations and T-cell-subsets in correlation to transection speed - Intraoperative blood loss and transfusion requirements |  | | --- | --- | | - Postoperative routine laboratory markers of liver damage (aspartate aminotransferase (AST), alanine aminotransferase (ALT)), and markers of liver function (bilirubin, prothrombin time) - Morbidity and mortality (Clavien-Dindo classification ) - Costs and health economics |  | |  |  | |  |  | |  |  | |

| Study Design |
| --- |
| Randomized controlled trial |

| Study Population |
| --- |
| Inclusion criteria:• Patients scheduled for elective major hepatic resection at the Department of General Surgery, Medical University of Vienna• Stapler hepatectomy and CUSA resection feasible based on preoperative imaging• Age equal or greater than 18 years• Informed consent |
| **Exclusion criteria** • Minor hepatectomy• Hepatitis B, Hepatitis C, HIV infection, autoimmune disease• Inflammatory conditions of the bowel such as Crohn's Disease • Pregnancy |

| Study Procedures |
| --- |
| 40 patients will be randomized in one of two treatment groups. The study will be conducted according to the Medical Product Law (§40 (3) MPG)  Randomization will be performed online by the Department of Medical Statistics and Informatics at the Medical University of Vienna (https://www.meduniwien.ac.at/randomizer/web/login.php)if resectability is confirmed by the surgeon.  Patients are blinded for the study intervention. An independent blinded investigator will assess the outcome measures.  **Group A: CUSA resection technique**  The liver parenchyma is divided along the transection line by CUSA (Cavitron ultrasonic aspirator; Valleylab, Boulder, CO) and bipolar forceps in a two surgeon technique. Vessels of less than 2 mm in diameter are coagulated with bipolar forceps. The remaining vessels are clipped or ligated. Hepatic veins and portal pedicles clamped and suture ligated. |
| **Group B: Stapler hepatectomy**  The liver parenchyma is crushed with a Pean clamp and subsequently divided using Covidien Endo-Gia™ Ultra Handle Short Staplers and Endo Gia™ TRI staple 60 mm or 45 mm AVM/AMT loading units (Covidien). Hepatic veins and portal pedicles clamped and suture ligated. |
| **Blood sampling timepoints:**   - pre surgery: systemic - pre resection (after liver mobilization): Portal vein, hepatic vein, systemic - post resection: Portal vein, hepatic vein, systemic - post surgery: systemic - postop day 1: systemic - postop day 3: systemic   => 400 blood samples  **Blood sampling sites:** Portal vein, hepatic veins, peripheral vein (systemic)  Blood samples will be collected in pre-chilled EDTA containing vacuum tubes. After centrifuging the samples at 1400 RPM (rounds per minute) for 10 min at 4°C, plasma will be stored at -80°C. Cytokine levels will be measured with multiplex ELISA assays (Aushon Biosystems).  **Evaluated markers:**   - MCP-1, MIP3-alpha, sCD163, Neopterin - pro- (INF-γ, IL-1β, IL-5, IL-6, IL12p70, TNFα) and anti-inflammatory (IL-4, IL-10, IL-13) cytokines - In patients undergoing hepatic resection for other than oncological reasons flow-cytometry staining will be performed according to standard protocol. PBMCs (peripheral blood mononuclear cells) will be stained with CD4, CD8, CD62L, CD69, FoxP3, CD127, CD45RO and CD45RA, to distinguish between the different T-cell subsets. |

| Efficacy Assessments |
| --- |
| **Primary endpoint:**  The transection time will be recorded by the anesthesiological team during surgery. The transection phase starts with opening the liver parenchyma after the transection line has been marked by electrocautery. It ends after complete division of the liver parenchyma. The cut surface of the resected liver will be photographed together with a 4 cm² reference scale in an exact 90° angle. The area of the liver transection surface will be calculated in cm² by setting the measured pixels of the cut surface in relation to the reference scale using Adobe Photoshop. The transection speed will expressed in cm²/min. |
| **Secondary endpoints:**  • Perioperative chemokine concentrations in correlation to transection speed  Cytokines and T-cell subsets are measured immediately before induction of anesthesia from a peripheral vein/artery, pre resection (after liver mobilization) from portal vein, hepatic vein, peripheral vein/artery, post resection from portal vein, hepatic vein, peripheral vein/artery, post surgery from peripheral vein/artery, postoperative day 1 from peripheral vein, postoperative day 3 from peripheral vein  • Intraoperative blood loss in ml  • Postoperative routine laboratory markers of liver damage (aspartate aminotransferase (AST), alanine aminotransferase (ALT)), and markers of liver function (bilirubin, prothrombin time) measured on first and third postoperative day  • Morbidity and Mortality according to the Clavien-Dindo classification within 30 days of surgery  • Costs and health economics will be calculated including used material, operation time, length of intensive care unit and hospital stay and associated morbidity requiring additional interventions. |

| Safety Assessments |
| --- |
| The rate of occurrence of perioperative safety findings potentially related to the applied surgical technique is one of the secondary endpoints of this study. Morbidity and Mortality will be classified according to the Clavien-Dindo classification : Grade DefinitionGrade I: Any deviation from the normal postoperative course without the need for pharmacological treatment or surgical, endoscopic, and radiological interventionsAllowed therapeutic regimens are: drugs as antiemetics, antipyretics, analgetics, diuretics, electrolytes, andphysiotherapy. This grade also includes wound infections opened at the bedsideGrade II: Requiring pharmacological treatment with drugs other than such allowed for grade I complicationsBlood transfusions and total parenteral nutrition are also includedGrade III: Requiring surgical, endoscopic or radiological interventionGrade IIIa: Intervention not under general anesthesiaGrade IIIb: Intervention under general anesthesiaGrade IV: Life-threatening complication (including CNS complications)* requiring IC/ICU managementGrade IVa: Single organ dysfunction (including dialysis)Grade IVb: Multiorgan dysfunctionGrade V: Death of a patientSuffix “d”: If the patient suffers from a complication at the time of discharge, the suffix “d” (for “disability”) is added to the respective grade of complication. This label indicates the need for a follow-up to fully evaluate the complication.*Brain hemorrhage, ischemic stroke, subarrachnoidal bleeding, but excluding transient ischemic attacks.CNS, central nervous system; IC, intermediate care; ICU, intensive care unit. |

| Safety Reporting |
| --- |
| All adverse events (AE) will be documented for each of the procedures. According to the Austrian law expedited reporting of serious AEs (SAE) is not required in a study not employing a medicinal product or medical device. Nevertheless SAEs will be listed in a yearly report to the Ethics committee and will be analyzed and presented in the final report (publication) |

| Insurance |
| --- |
| A patient insurance was taken out at the Zurich; policy nb: 07229622-2 |

| Data Quality |
| --- |
| The investigator or a designated representative enters all protocol-required information in the case report form (CRF) immediately after data acquisition.  Independent, continuous monitoring including source data verification is not required according to the Austrian law. However, SAEs and Informed consent and the primary efficacy variable will be checked by an independent person not involved in the study.  Quality assurance: Audits are not scheduled for this study. |

| Statistical Analysis |
| --- |
| Sample size calculation: 20 patients per group  In a comparison of clamp-crush with vascular occlusion (comparable to speed of stapler hepatectomy) and CUSA resection the mean difference in transection speed was 1.60 cm^2^/min (95% CI 0.89 to 2.31). The transection speed was 3.9 ± 0.3 SEM for clamp crush and 2.3 ± 0.2 SEM for CUSA resection. SD was 1.5 for clamp crush and 1 for CUSA . Based on this, each group would need 12 patients for an alpha: Prob (reject H_0_ when H_0_ is true) of 0.05 and power of 80%: Prob (reject H_0_ when H_1_ is true) of 0.80. If we assume a slightly longer transection time for stapler hepatectomy and a drop out quote of 15%, 20 patients per group seem adequate. |
| The primary efficacy variable will be analyzed with a t-test for independent samples. A p-value of <0.05 will be the threshold for significance.  Secondary endpoints will be assessed descriptively only. |
|  |

| Timetable |
| --- |
| **Duration:** 24 months |

**Submitted by**

**Dr. Christoph Schwarz**

**& Prof. Dr. Klaus Kaczirek**

**Medizinische Universität Wien**

**Univ. Klinik für Chirurgie, Abteilung für Allgemein-, Gefäß- und Transplantchirurgie**

**References**

1. Gurusamy KS, Pamecha V, Sharma D, Davidson BR. Techniques for liver parenchymal transection in liver resection. Cochrane Database Syst Rev 2009:CD006880.

2. Jarnagin WR, Gonen M, Fong Y, et al. Improvement in perioperative outcome after hepatic resection: analysis of 1,803 consecutive cases over the past decade. Ann Surg 2002;236:397-406; discussion -7.

3. Rahbari NN, Elbers H, Koch M, et al. Clamp-Crushing versus stapler hepatectomy for transection of the parenchyma in elective hepatic resection (CRUNSH) - A randomized controlled trial (NCT01049607). BMC Surg 2011;11:22.

4. Tamandl D, Gruenberger B, Herberger B, et al. Surgery after neoadjuvant chemotherapy for colorectal liver metastases is safe and feasible in elderly patients. J Surg Oncol 2009;100:364-71.

5. Tamandl D, Gruenberger B, Klinger M, et al. Liver resection remains a safe procedure after neoadjuvant chemotherapy including bevacizumab: a case-controlled study. Ann Surg 2010;252:124-30.

6. Kimura F, Shimizu H, Yoshidome H, et al. Circulating cytokines, chemokines, and stress hormones are increased in patients with organ dysfunction following liver resection. J Surg Res 2006;133:102-12.

7. Yamauchi H, Kobayashi E, Yoshida T, et al. Changes in immune-endocrine response after surgery. Cytokine 1998;10:549-54.

8. Cruickshank AM, Fraser WD, Burns HJ, et al. Response of serum interleukin-6 in patients undergoing elective surgery of varying severity. Clin Sci (Lond) 1990;79:161-5.

9. Baigrie RJ, Lamont PM, Kwiatkowski D, et al. Systemic cytokine response after major surgery. Br J Surg 1992;79:757-60.

10. Mokart D, Merlin M, Sannini A, et al. Procalcitonin, interleukin 6 and systemic inflammatory response syndrome (SIRS): early markers of postoperative sepsis after major surgery. Br J Anaesth 2005;94:767-73.

11. Jawa RS, Anillo S, Huntoon K, et al. Interleukin-6 in surgery, trauma, and critical care part II: clinical implications. J Intensive Care Med 2011;26:73-87.

12. Zhai Y, Busuttil RW, Kupiec-Weglinski JW. Liver ischemia and reperfusion injury: new insights into mechanisms of innate-adaptive immune-mediated tissue inflammation. Am J Transplant 2011;11:1563-9.

13. Caldwell CC, Tschoep J, Lentsch AB. Lymphocyte function during hepatic ischemia/reperfusion injury. J Leukoc Biol 2007;82:457-64.

14. Dindo D, Demartines N, Clavien PA. Classification of surgical complications: a new proposal with evaluation in a cohort of 6336 patients and results of a survey. Ann Surg 2004;240:205-13.

15. Lesurtel M, Selzner M, Petrowsky H, et al. How should transection of the liver be performed?: a prospective randomized study in 100 consecutive patients: comparing four different transection strategies. Ann Surg 2005;242:814-22, discussion 22-3.
